# Protoss: a holistic approach to predict tautomers and protonation states in protein-ligand complexes

**DOI:** 10.1186/1758-2946-6-12

**Published:** 2014-04-03

**Authors:** Stefan Bietz, Sascha Urbaczek, Benjamin Schulz, Matthias Rarey

**Affiliations:** 1 Center for Bioinformatics(ZBH), Universität Hamburg, Bundesstr. 43, 20146 Hamburg, Germany; 2 Current address: BioSolveIT GmbH, An der Ziegelei 79, 53757 St. Augustin, Germany

**Keywords:** Protein-ligand complex, Tautomers, Protonation states, Hydrogen placement

## Abstract

The calculation of hydrogen positions is a common preprocessing step when working with crystal structures of protein-ligand complexes. An explicit description of hydrogen atoms is generally needed in order to analyze the binding mode of particular ligands or to calculate the associated binding energies. Due to the large number of degrees of freedom resulting from different chemical moieties and the high degree of mutual dependence this problem is anything but trivial. In addition to an efficient algorithm to take care of the complexity resulting from complicated hydrogen bonding networks, a robust chemical model is needed to describe effects such as tautomerism and ionization consistently. We present a novel method for the placement of hydrogen coordinates in protein-ligand complexes which takes tautomers and protonation states of both protein and ligand into account. Our method generates the most probable hydrogen positions on the basis of an optimal hydrogen bonding network using an empirical scoring function. The high quality of our results could be verified by comparison to the manually adjusted Astex diverse set and a remarkably low rate of undesirable hydrogen contacts compared to other tools.

## Background

Crystal structures of protein-ligand complexes play an important role in the drug development process. They provide valuable insights into where and how molecules interact with their respective target proteins and thus are the basis for further optimization strategies. They also serve as starting point for numerous structure-based in-silico techniques such as molecular docking or pharmacophore generation. Furthermore, the statistical analysis of large collections of crystal structures is a common means to gain general knowledge about molecular interactions and geometry. These results are often used to derive parameters for various computational methods. All of the above-mentioned applications depend on information about the interactions between protein and molecules with hydrogen bonds being one of the most important types. Due to insufficient resolution, the vast majority of the entries in the Protein Data Bank (PDB) [1] only contain coordinates of non-hydrogen atoms. In order to be able to work with these entries, automated procedures for the placement of hydrogen atoms are needed. Considering its importance, it is not surprising that a large number of different methodologies have been developed to tackle this task. A thorough review of these different approaches has been given by Forrest and Honig [2].

While many of these applications show substantial differences concerning their subjective function or their underlying optimization algorithms, most of them share the degrees of freedom which are used to tackle the uncertainties of structure determination [3-8]. Typically, these comprise rotatable hydrogens, tautomers and protonation states of particular amino acids, alternative water orientations, and terminal side chain flips. Indeed, this covers the most important ambiguities of protein structures, but neglects crucial aspects of ligand molecules. Different tautomers and protonation states can lead to substantially different interaction patterns. Hence, considering alternative ligand states has a high impact on the quality of hydrogen bonding networks, especially for applications dealing with ligand binding. Neglecting these degrees of freedom might easily lead to erroneous predictions, including the omission of relevant hydrogen bonds and the generation of hydrogen clashes. Nevertheless, targeting this problem has not drawn much attention in the literature yet. This might be reasoned in a deviating focus of most hydrogen prediction tools, which concentrate rather on the whole protein than on single binding sites, but it might also reflect the difficulty of properly modeling complex phenomena like tautomerism and ionization of arbitrary organic molecules. However, some of the more recently developed methods consider these effects at least to some extent.

Protonate 3D [9,10] has been developed for the prediction of hydrogen coordinates as a preprocessing step to structure-based computational applications, e.g. protein-ligand docking or molecular dynamics. Beside well-established degrees of freedom for protein side chains, it is also capable of considering selected alternative states of other chemical groups. This is technically realized by a SMARTS [11]/SMILES [12]-based template collection stored in a predefined parameter file which must explicitly contain all tautomeric and protonation states that should be considered for a specific chemical group. Furthermore, Protonate 3D uses a prioritizing branch-and-bound algorithm in combination with a preceding dead-end elimination to handle the state space optimization problem and a force field based energy model including additional terms for tautomerism and ionization effects.

The modeling and simulation suite YASARA [13,14] provides a sub-module for the prediction of hydrogen coordinates which is able to consider alternative protonation states and tautomers of non-protein-like chemical substructures. Similarly to Protonate 3D, a configuration file contains template definitions for different potential states of these substructures represented as SMILES strings. Its default collection of considered substructures is a little more comprehensive, but its generality is still limited by the fact that all molecular states have to be explicitly defined. The optimization problem is tackled with an algorithm, originally developed for side chain prediction, which combines a dead-end elimination, a branch-and-bound backtracking, and a graph decomposition approach [15]. Interestingly, the underlying empirical scoring model, in contrast to most other hydrogen prediction tools, targets a minimization of the amount of unsaturated hydrogen bond donors or acceptors instead of a maximization of the number of attractive interactions.

We present a novel method for the placement of hydrogen coordinates in protein-ligand complexes. By using the consistent chemical description provided by the NAOMI model [16], tautomeric and protonation states of both protein and ligand are handled consistently. The method is a substantial extension of Protoss [17] which has been developed earlier. The optimal hydrogen bonding network is determined on the basis of the quality of all possible hydrogen bonds in combination with the stability of the involved chemical groups. There is to the best of our knowledge no other method described in the literature which is able to handle the degrees of freedom for protein and ligand in an comparable generality.

## Methods

The purpose of the presented method is the generation of the most probable hydrogen placement for a given protein-ligand complex. The underlying optimization procedure is based on an empirical scoring scheme designed to identify an optimal hydrogen bonding network. This scheme takes both the quality of hydrogen bond interactions and the relative stability of different chemical species into account. The procedure is performed in separate steps which will be explained in detail in the following sections. Due to the exceptional importance of the PDB as source for input structures, we have added a subsection in which the necessary preprocessing steps for working with PDB files are discussed.

### Input from PDB files

In contrast to most other chemical file formats, the PDB format [18] does not include any information about bond orders or atom types so that these properties must be derived directly from the provided atomic coordinates. In case of biological macromolecules, e.g., proteins, this process can be considerably facilitated by using structural templates for standard residues. The necessary data for both the subdivision of proteins into residues and the identification of particular atoms is provided in the coordinate section of the PDB format. In case of incomplete residues, the missing atoms are topologically added in order to ensure an accurate description. They will, however, not have valid coordinates and are thus ignored during the calculation of interactions. For the large and steadily growing number of different small molecules in the PDB, predefined structural templates are generally not a viable option. In this case a generic method for the construction of molecules from three-dimensional coordinates is needed. This evidently also applies to non-standard residues for which no predefined template is available. We use a method based on the NAOMI model for that purpose [19]. Both strategies eventually result in isolated components which have to be connected in order to build the complete protein structure. The connection of standard residues with peptidic bonds is again handled with recourse to predefined templates. All other types are based on a procedure similar to that used for the generic construction from three-dimensional coordinates. The only difference is that the method is applied to a substructure rather than the complete molecule. In this way the consistent description of molecules can be used to reliably handle the integration of residues into proteins. The description of both proteins and molecules is based on the NAOMI model, meaning that consistent atom type and bond order information is available throughout the next steps.

### Initial hydrogen positions

Initial hydrogen coordinates are calculated on the basis of idealized geometries provided by the atom types of the NAOMI model. These geometries reflect the hybridization states of the respective atoms and are based on the general concepts of VSEPR theory [20]. In combination with the coordinates of the covalently-bound non-hydrogen atoms, knowledge about the atom’s hybridization state can be used to calculate reasonable positions for hydrogens. The concrete orientation of the respective hydrogen bonds is in many cases unambiguously determined by the constraints imposed by the atom’s local geometry. In case of an sp3 hybridized carbon atom with three non-hydrogen bonds, for instance, the direction of the bond coincides with the connection line to the unoccupied vertex position of the underlying tetrahedron. There are, however, a few cases for which multiple acceptable orientations exist. The most prominent examples in protein-ligand complexes are isolated atoms (e.g. water), terminal atoms (e.g. alcohols, acyclic amines) and particular types of ring atoms (e.g. cyclic secondary amines). In these cases the orientation of hydrogens cannot be unambiguously derived from the heavy atom skeleton of the respective molecule. The final decision can only be made under consideration of all chemical moieties in close vicinity so that only preliminary positions can be calculated at this point. Another type of ambiguity arises with respect to the initial tautomeric and ionization states of both residues and ligands as these will obviously influence the corresponding hydrogen positions. For this purpose the normalization procedures described in [21] are applied prior to the generation of initial hydrogen coordinates. Free amino and acid groups of residues resulting from chain breaks are a special case. If the PDB file does not indicate that these residues are in fact terminal, they will be treated internally as incomplete parts of an amide bond and thus kept in their neutral state. At the end of the procedure, each hydrogen atom in the protein will have three-dimensional coordinates which are in accordance with the hybridization states of the respective bond partners. In case of multiple alternatives, these preliminary positions, however, are just needed for technical reasons and will be adapted in later steps.

### Enumeration of alternative hydrogen positions

**Figure 1:**
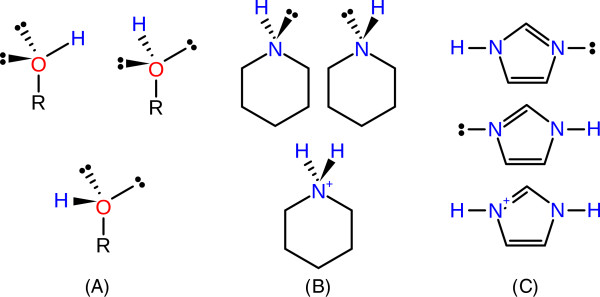
**Three examples for VMRs with alternative hydrogen positions and free electron pairs.** Primary alcohols **(A)** are considered as rotatable and the associated hydrogen atom can occupy any position on the orbit around the oxygen atom. Three exemplary orientations are shown. Cyclic secondary amines **(B)** can either be protonated and positively charged or neutral. In the latter case the hydrogen atom can occupy two distinct positions. The imidazole ring **(C)** can either occur as one of the two different tautomeric species or in its ionized form. In contrast to the other examples the VMR contains multiple atoms in this case.

Based on the initial assignment of hydrogen positions, tautomers and protonation states, substructures with variable hydrogen positions in both protein and ligand are identified. The considered types of variability are rotations of terminal hydrogen atoms, potential side-chain flips for specific residues, alternative tautomeric forms, different protonation states, and alternative orientations of water molecules. For each substructure, all different placements of hydrogen atoms, called alternative modes in the following, are enumerated (see Figure 1 for examples). In contrast to the previously published Protoss version, tautomeric and protonation states for small molecules and non-standard residues are also taken into account. These are generated using the valence state combination model presented in a separate publication [21]. Since the details of these calculations are beyond the scope of the presented method, we will only give a short overview with focus on those aspects relevant in the current context. The workflow starts with the partitioning of the molecule into non-overlapping substructures which correspond for the most part to conjugated ringsystems and functional groups. In some cases substituents, e.g., alcohols and amines, are considered as part of a ringsystem as they are necessary for the consistent generation of tautomers. The partitioning is retained throughout the following steps as it reflects the dependency between the hydrogen positions for the atoms in the substructures. These will be referred to as Variable Mode Regions (VMR) in the following. Protonation states and tautomers are enumerated for each VMR individually and stored in form of a list containing the alternative modes together with an integer-based score. These scores provide an order of preference which is crucial when deciding if the default mode of a VMR should be changed in order to optimize the hydrogen bonding network. The underlying scoring scheme is based on the identification of predefined structural fragments in the respective modes of the VMRs. Each fragment corresponds to a different tautomeric form or protonation state and is associated with a partial score. The total score of the mode is calculated as the sum of these individual contributions. In case of ringsystems the score comprises contributions from each ring and its respective substituents. Scores for functional groups are either generated by matching the whole group directly, which is the usual case, or by partitioning the group into subgroups and adding the scores of the smaller subgroups. The values for the individual contributions of the respective substructures have been derived from different pairs of tautomers for which the preference was experimentally known and from *pK*_*a*_ tables.

### Hydrogen bond interactions

**Figure 2:**
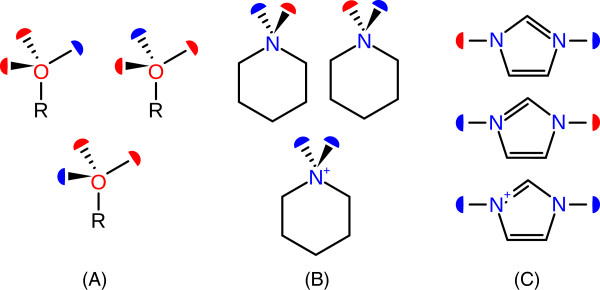
**Three examples for VMRs with alternative modes.** Donor surfaces are represented by blue half-circles and acceptor surfaces are represented by red half-circles. In the first two examples, primary alcohol **(A)** and cyclic secondary amine **(B)**, each mode comprises multiple interaction surfaces for a single atom. In case of the imidazole ring **(C)** the respective surfaces are associated with different atoms.

Since Protoss is designed to identify the best hydrogen bonding network, it requires structural information for the evaluation of potential polar interactions. Therefore, each mode is internally represented as a set of interaction surfaces, originally developed in the context of molecular docking [22]. This is shown in Figure 2 (A) for the straightforward case of a rotatable hydroxyl group. Each mode includes one interaction surface associated with the orientation of the hydrogen atom (donor surface) and two additional interaction surfaces associated with the atom’s free electron pairs (acceptor surface). The modes for a secondary amine are shown in Figure 2 (B) in order to exemplify the handling of protonation states. In this case the number of donor and acceptor surfaces of each mode is not necessarily identical. Modes for tautomeric states introduce a higher complexity since they involve hydrogen positions at multiple atoms simultaneously. The corresponding modes for an imidazole moiety are shown in Figure 2 (C). In this case, only specific combinations of interaction surfaces are considered reflecting the different tautomeric states of the molecule. These combinations are derived from the alternative modes for the VMRs generated in the previous step.

The objective function for the evaluation of the hydrogen bonding network comprises, as in the previous Protoss version, the analysis of hydrogen bonds as well as metal interactions. In order to prevent the generation of undesirable contacts of polar groups in the protein-ligand interface, the scoring function has been extended by an additional term for the assessment of repulsive contributions such as donor-donor, donor-metal, or acceptor-acceptor contacts. The interaction quality is for both cases, attractive and repulsive interactions, determined by a geometric criterion which measures the relative orientation of two interaction surfaces (see [22]). However, in contrast to hydrogen bonds and metal interactions, repulsions have naturally a destabilizing influence on the total energy of the hydrogen bonding network.

### Optimization procedure

The optimization procedure is based on two main aspects, namely the scoring of hydrogen bond interactions and the resolution of dependencies in the hydrogen bonding network. The latter is represented by a graph structure in which each node corresponds to a single VMR with all its associated alternative modes. Edges between nodes are formed if there exists at least one relevant interaction between the atoms of the respective VMRs. This is determined by a geometric criterion. In the first step, the scoring phase, the alternative modes of each node are assigned a base score which is composed of an intrinsic stability contribution reflecting the preference of the respective tautomeric form or protonation state it represents and a term for the interaction energies with all non-variable parts of the complex. The value of the stability contribution is derived from the score calculated by the generic scoring scheme described above. Each edge contains a matrix that stores an interaction score for each combination of modes of its two incident nodes. In the second step, the optimization phase, a combination of a cycle decomposition and a dynamic programming algorithm is used to find an optimal hydrogen bonding network by minimizing the total score and selecting a distinct mode for every VMR. For a set of selected modes *M*, the total score is therefore calculated by Equation 1.

(1) $$\begin{array}{l} \text{totalScore}(M)=\underset{m\in M}{\sum }\text{baseScore}(m)\\ \;+\underset{m,n\in M}{\sum }\left(\text{interactions}(m,n)+\text{repulsions}(m,n)\right) \end{array}$$

Further details about the optimization procedure can be found in a previous publication [17]. Finally, the optimized coordinates of all variable hydrogen atoms are generated by transferring the structural information of the individual modes back onto the protein-ligand complex.

## Results and discussion

### Tautomeric frequencies

Most hydrogen prediction tools for protein-ligand complexes only handle tautomerism for moieties from proteinogenic amino acids or by explicit lists of substructure transformation rules. In order to demonstrate the insufficiencies of this approach, we counted all substructures contained in the Ligand Expo database (accessed Jan 3, 2014) [23], for which we were able to identify sensible alternative tautomers or protonation states. Furthermore, we split the set into two groups. First, the set of functional groups which also appear in protein side chains, namely carboxylates, primary amines, and imidazoles (classical VMRs). Second, all other functional groups and conjugated substructures for which more than one sensible state could be created (advanced VMRs). Rotational degrees of freedom were neglected for this analysis.

We found that only 19% of the Ligand Expo database molecules did not show any VMR with alternative tautomers or protonation states. Furthermore, 17% of all molecules only contain substructures from the classical VMRs set. For all other molecules, at least one advanced VMR was observed.

**Figure 3:**
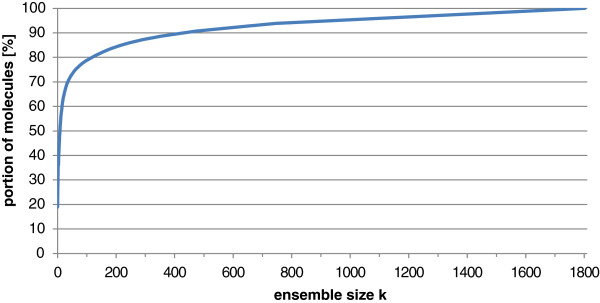
**Dependency of the portion of Ligand Expo molecules whose protonational variability can be completely described by a set of the** ***k* most frequent VMRs on the ensemble size** ***k*.**

Overall, we found 1802 structurally different, canonical VMR types. In order to analyze the relevance of these different substructures, we first sorted the list of VMRs according to the portion of molecules containing the respective VMR and then plotted the amount of molecules whose variability with respect to tautomerism and protonation can be completely described by a set of the *k* most frequent VMRs (see Figure 3). The results show that, e.g., a set of approximately 430 substructures is required to consider the full variability for 90% of all molecules in the Ligand Expo database. In general, the curve progression clearly illustrates the strong dependency of low prediction error rates on the consideration of a wide range of chemical substructures.

**Figure 4:**
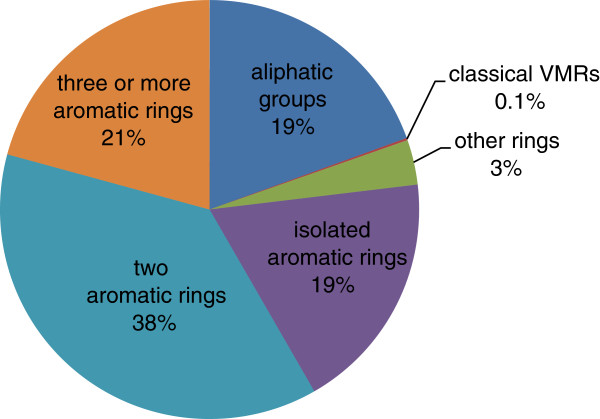
Relative amount of structurally different VMR types in the Ligand Expo database.

Figure 4 additionally depicts the absolute amount of different VMRs for various chemical classes. This classification demonstrates that the high amount of different VMRs is mostly reasoned in the diversity of aromatic substructures. The difficulty of correctly treating more complicated substructures, such as annulated aromatic ringsystems, motivates a generic approach for handling tautomerism.

### Undesirable contacts

One of the primary requirements on hydrogen placement is to avoid the generation of undesirable contacts such as close donor-donor, donor-metal or acceptor-acceptor interactions. In order to evaluate the effect of considering alternative protonation and tautomeric states on this issue, we analyzed the occurrence of undesirable contacts in the results of the hydrogen prediction tools Protonate 3D (as implemented in MOE 2013.08 [10]), YASARA (version 13.9.8 [14]), and Protoss. The latter was used in two alternative versions, with and without an analysis of alternative tautomers and protonation states. Apart from that, all tools were applied with default settings. The sc-PDB database v.2012 [24] served as basis for this test, as it constitutes a comprehensive and diverse database of pharmacological relevant protein-ligand complexes. However, as the protein files provided by the sc-PDB do not contain water molecules, we used the corresponding original files from the PDB instead. The sc-PDB v.2012 consists of all in all 8077 protein-ligand complexes. Nine of them were not available in the PDB anymore (November 2013) and have therefore been excluded. The remaining 8068 structures were further processed by a clean-up step removing all existing hydrogen atoms, atom duplicates, and overlapping entities in order to reduce possible error sources which might bias the hydrogen prediction and validation experiments. This procedure comprises a series of atom entry filtering steps which were processed in the following order. First, all hydrogen atom entries were erased. Second, all residue entries were identified that overlap with the reference ligand. In this and all following cases, an overlap was defined as an atom distance of equal or less than 1 Å. Furthermore, an overlapping residue entry was defined to represent a part of the reference ligand if for each of its atom entries the closest atom of the reference ligand has a maximum distance of 1 Å and the same chemical element type. (This rather fuzzy matching criterion was chosen because some sc-PDB ligands are shifted or have a slightly different conformation compared to the original PDB structure). Otherwise the overlapping entry was removed. If an overlapping residue entry contains alternate locations we only kept that conformation which fits the reference ligand best. In case that the best conformation does not fulfill the matching criterion, the residue entry was only retained if the first alternate location has no overlap with the reference ligand. In the third step, all other atom entries were checked for alternate locations and only the first position per atom was kept. In a final step, all residue entries were dropped, which overlap with any preceding entry in the file or exhibit an internal atom overlap.

In 27 cases this cleanup procedure led to a partial or total removal of the reference ligand’s heavy atoms, e.g. if the sc-PDB ligand, compared to the original PDB structure, exhibits a different conformation, deviating element, additional atoms, or an internal atom overlap. Therefore these structures were also removed.

**Figure 5:**
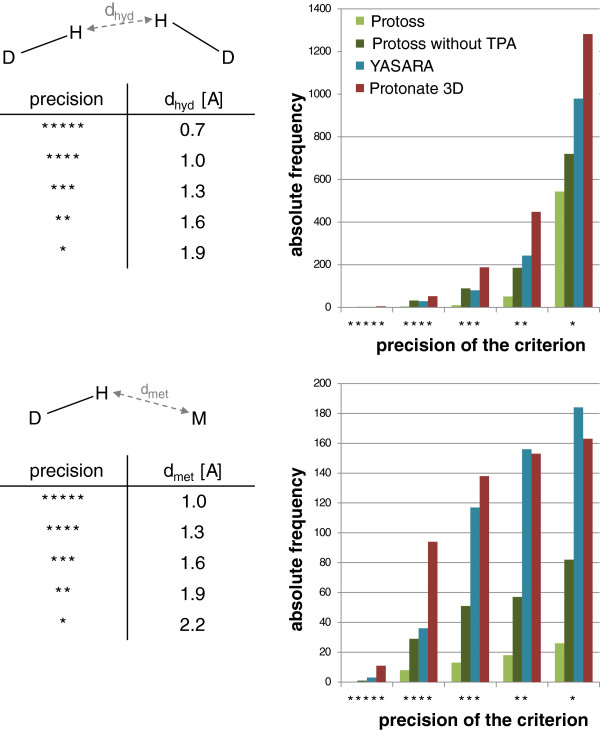
**Frequencies of undesirable contacts in the predictions of YASARA, Protonate 3D and Protoss on the basis of a series of pure distance criteria.** A contact is considered undesirable, if the distance between the respective atoms falls under the threshold given in the table on the left. The interaction schemes illustrate donor (D), hydrogen (H), and metal (M) atoms as well as the measured distances.

**Figure 6:**
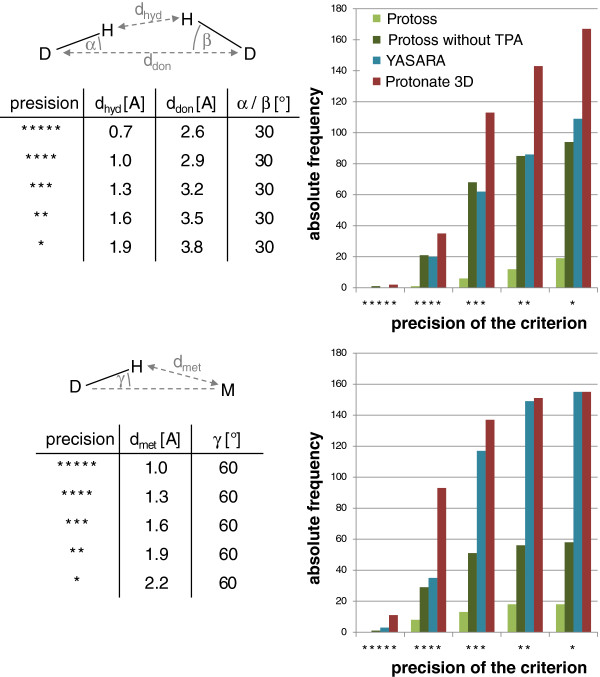
**Frequencies of undesirable contacts in the predictions of YASARA, Protonate 3D and Protoss on the basis of series of combined angle/distance criteria.** A contact is considered undesirable, if all four measured values fall under the thresholds given in the table on the left. The interaction schemes illustrate donor (D), hydrogen (H), and metal (M) atoms as well as the measured distances and angles.

For the remaining set of 8041 files, all three tools were used to add new hydrogen atoms and to optimize the hydrogen bonding networks. As the Yasara version used in this study shuts down during the prediction for one complex (3ptq), this structure was also excluded. Eventually, the results were scanned for undesirable contacts, which were defined as follows: All oxygen and nitrogen atoms of the ligand or the active site (6.5 Å around the ligand) which have at least one hydrogen bound were considered as hydrogen bond donors. Two hydrogen bond donors are defined to form an undesirable contact if the hydrogen atom distance is equal or less than a certain threshold. Likewise, an undesirable contact between a donor and a metal ion is determined on the basis of the hydrogen-metal distance (see Figure 5). For both cases, exactly one of the counterparts had to be part of the ligand. Beside this simple distance criteria, we also analyzed both types of contacts under consideration of additional measures, namely the heavy atom distance and the angles formed by both heavy atoms and one of the hydrogens (see Figure 6). We also defined different threshold sets to investigate the dependency of the error frequency on the precision of the interaction criterion. All used precision levels and their respective thresholds are listed in the tables in Figure 5 and Figure 6. Although an additional investigation of acceptor-acceptor contacts could provide further insights, we explicitly avoided this analysis, because acceptor orientations cannot be analyzed without interpreting the input data on the basis of geometric assumptions of an internal chemical model, which would compulsorily influence the evaluation. Overall, the possibly most conspicuous and expected finding is that the error frequency increases with decreasing precision of the interaction criterion. This effect can be observed for all prediction tools. The higher rate of undesirable contacts for the Protoss version without tautomer analysis throughout all precision levels clearly demonstrates the benefit of considering tautomerism and protonation states for the performance of hydrogen prediction.

### Comparison to manual adjustment

Ultimately, a hydrogen prediction tool should be validated against experimental data. Unfortunately, there is only a very limited amount of experimental data that might be used for such an evaluation due the difficulties of determining hydrogen coordinates with X-ray crystallography.

As a result of the insufficient amount of experimental data, we intend to demonstrate the properness of our approach on the basis of the Astex diverse set [25] (Astex Set). This collection of 85 protein-ligand complexes, which was developed for the validation of docking performance, contains ligands which are manually adjusted with respect to their protonation and tautomeric states. Therefore, the Astex Set seems to be suitable for a verification of predicted ligand states. For each target structure in the dataset, the original file was retrieved from the PDB, preprocessed as described in the previous section (removing existing hydrogen atoms, atom duplicates, and overlapping entities) and given to Protonate 3D, YASARA, and Protoss for generating new hydrogens as well as their coordinates. The results were then written to PDB files and compared to the ligand topology given in the Astex Set.

The topological ligand comparison was realized by a simple string comparison of Unique SMILES [26]. However, as the bond orders of the internal molecule representation that was used for the Unique SMILES generation are derived from PDB files, there is still a theoretical risk of misinterpreting the molecular topology. Therefore, all automatically detected deviations where additionally confirmed by visual comparison to the graphical molecular representations of the respective tools.

The deviating solutions are classified according to the deviation type, thus whether the solution constitutes a different tautomer, protonation state, or redox form. Furthermore, the quality of the hydrogen bonding network with respect to undesirable contacts and missing a hydrogen bonds is analyzed. Since a different redox form constitutes a more serious problem, the latter aspect is only evaluated for deviating tautomers and protonation states. In contrast to erroneous redox forms, deviating protonation or tautomeric states are not necessarily incorrect. However, a worse hydrogen bonding network is at least a strong hint that the respective structure is inferior. A hydrogen bond was defined by a maximum heavy atom distance of 3.5 Å and a minimal donor-hydrogen-acceptor angle of 150°. Undesirable contacts were defined on the basis of precision level 2 (**) (see Figure 5).

**Figure 7:**
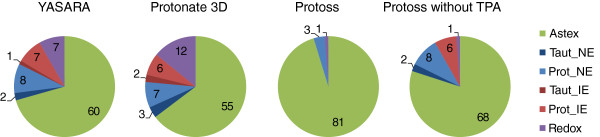
**Classification of the prediction results for YASARA, Protonate 3D, Protoss, and Protoss without tautomer and protonation state analysis (TPA) on the Astex Set.** Absolute fractions of all 85 Astex Set complexes are shown. The classification is depending on whether the ligand state is accordant to the Astex Set reference or represents a different tautomeric state without interaction errors (Taut_NE), a different protonation state without interaction errors (Prot_NE), a different tautomer or protonation state exhibiting interaction errors (Taut_IE / Prot_IE) or an incorrect redox form (Redox).

Figure 7 illustrates the amount of accordant and deviating ligand states as well as the five classes of the deviating solutions. For all of the three hydrogen prediction tools, the set of proposed solutions which are in accordance with the ligand states in the Astex Set (depicted in green) constitutes the major portion. A closer look to the fractions of different tautomers and protonation states which form less interactions or even undesirable contacts (light and dark red), as well as incorrect redox forms (purple) demonstrate the importance of a comprehensive initialization of ligand molecules. A comparison to the Protoss version which does not execute an analysis of tautomers and protonations state (TPA) demonstrates the reduction of critical cases and hence, also the ability of resolving erroneous prediction performance.

**Figure 8:**
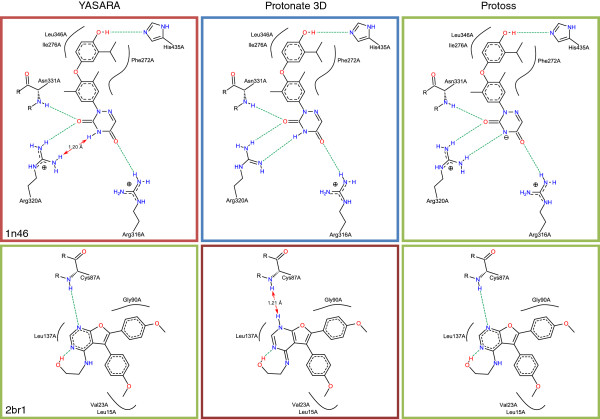
**Exemplary comparison of YASARA, Protonate 3D and Protoss predictions on two complex structures from the Astex Set (PDB codes 1n46 and 2br1).** Hydrogen bonds are depicted as green dashed lines. Undesirable contacts are indicated by red arrows. The frame coloring corresponds to the classification depicted in Figure 7.

The classification is also illustrated by the following case studies taken from the Astex Set. Given the complex of the human thyroid receptor beta ligand-binding domain and its 6-azauracil-based ligand from PDB structure 1n46 [27], Protoss proposes a negatively charged state of the azauracil moiety which is able to form three hydrogen bonds with the surrounding arginine residues (see Figure 8). This prediction is in accordance with the ligand state given by the Astex Set. In contrast to this, Protonate 3D chooses a neutral ligand state and deprotonates Arg320 instead. Although this leads to the same number of hydrogen bonds, considering the *pK*_*a*_ values of 6-azauracil (*pK*_*a*_=6.9 [28]) and the protonated arginine side chain (*pK*_*a*_=12.5 [29]) this solution seems to be less likely. YASARA neither deprotonates the azauracil moiety nor the guanidinium of Arg320 which leads to the loss of a hydrogen bond and simultaneously to the formation of a close donor-donor contact with a hydrogen distance of 1.20 Å. Figure 8 also depicts the solutions for serine/threonine-protein kinase Chk1 complexed with a furanopyrimidine inhibitor (2br1) [30]. While both Protoss and YASARA successfully reproduce the state of the reference ligand, which is stabilized by a hydrogen bond to the backbone of Cys87 and an internal interaction with a hydroxyl group, Protonate 3D selects a different tautomer. Thereby, the hydrogen bond to Cys87 is replaced by a contact of two donors with a hydrogen distance of 1.21 Å.

**Figure 9:**
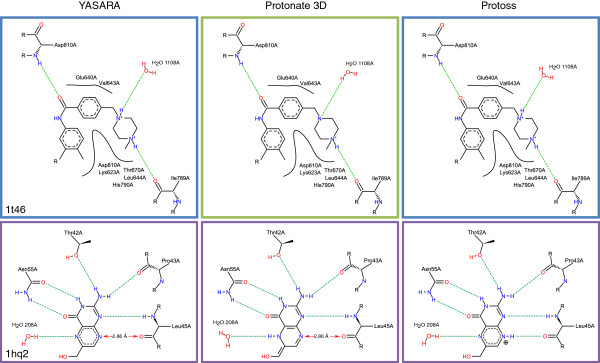
**Exemplary comparison of YASARA, Protonate 3D and Protoss predictions on two complex structures from the Astex Set (PDB codes 1t46 and 1hq2).** Hydrogen bonds are depicted as green dashed lines. Undesirable contacts are indicated by red arrows. The frame coloring corresponds to the classification depicted in Figure 7.

All in all there are only four cases where Protoss produces a ligand state that differs from the reference given by the Astex Set. However, we did not observe a missing hydrogen bond or an undesirable contact in any of these binding sites. For an inhibited thrombin complex (1oyt, not shown) [31] Protoss proposes a protonated nitrogen in contrast to a neutral state in the Astex Set. However, this does not change the quality of the hydrogen bonding network since this atom is not involved in a polar interaction. In case of an adenosine deaminase structure complexed with a non-nucleoside inhibitor (1uml, not shown) [32], Protoss protonates an imidazole ring of the ligand, which enables the formation of a hydrogen bond to Asp296. The same interaction can be found in the Astex Set structure, though here the hydrogen is located at Asp296 instead. In another example, shown in Figure 9, Protoss chooses a double protonated state of a piperazine ring (1t46) [33]. This can be explained by the fact that only conjugated ring systems are handled as a unit, while polar groups in others rings are treated separately. Here, only Protonate 3D identifies the more likely single protonated state. YASARA also predicts the double charged piperazine ring. However, as one of the piperazine nitrogen only interacts with a water molecule, this deviation has no significant effect on the hydrogen bonding network.

**Figure 10:**
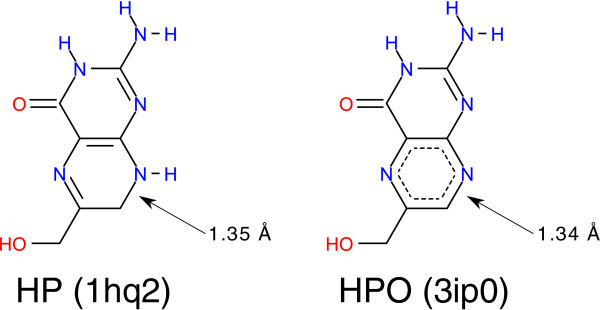
Structure diagrams of 6-Hydroxymethyl-7,8-dihydropterin (HP) and 6-Hydroxymethylpterin (HPO).

The only critical solution produced by Protoss constitutes the complex of E.coli 6-Hydroxymethyl-7,8-dihydropterin pyrophosphokinase and its substrate. For this target, all three tools fail to produce the correct redox form of the ligand. This might be reasoned in the exceptionally short bond length of carbon C7 and nitrogen N8 with a distance in the PDB file of 1.35 Å (1hq2) [34]. Interestingly, there is another PDB structure of the same complex which contains the oxidized form of the ligand (3ip0) [34]. Here, the same bond has a length of 1.34 Å (see Figure 10). In this case, it is obviously a tough task to predict the correct redox form automatically only on the basis of heavy atom coordinates.

### Computing time

On average, the hydrogen prediction by Protoss took 2.47 seconds for a complex from the Astex Set. The median of this prediction series is 0.93 seconds. This includes file IO, preprocessing, and hydrogen bonding network optimization for the whole protein-ligand complex with all ligands, co-factors and water molecules. All runtime measurements were performed on a single core of an Intel Core i7-2600 with 3.4 GHz and 8 GB of memory.

## Conclusion

There are several known cases in which a small change in the ligand molecule, resulting in a single additional hydrogen bond, makes a huge difference in binding affinity. Therefore, the correct assignment of the ligand’s tautomeric form, its protonation state and hydrogen orientations is a mandatory step in structure-based molecular design. Especially precise protein-ligand scoring functions, as a key component in docking and lead optimization procedures, rely on a correct protonation. Since validation procedures for docking and scoring are mostly based on carefully, hand-prepared test cases, the influence of wrong tautomerism and protonation is quickly overseen.

Several methods exist already addressing this important preprocessing step, however, most approaches lack a comprehensive model of ligand tautomerism. Here, we present a novel method for the placement of hydrogen coordinates in protein-ligand complexes under consideration of both tautomeric and protonation states. The method implements an optimization procedure designed to identify the best hydrogen bonding network based on a generic scoring function. Its main application is the automatic preparation of protein binding sites for structure-based virtual screening and large-scale statistical analysis of molecular interactions in biological systems. Our validation studies show that for this purpose our approach yields results which are in good agreement with manually adjusted ligand states. Numerous case studies demonstrate that the resulting molecular states are both comprehensible and chemically reasonable.

## Availability

Protoss is available free of charge for academic use as a web service at http://www.zbh.uni-hamburg.de/protoss.

## Competing interests

The authors declare that they have no competing interests.

## Authors’ contributions

SB and SU contributed equally to this work. They developed the algorithmic concepts, implemented the software, tested it, and prepared the manuscript. BS contributed to the implementation and testing of Protoss. MR initiated the development and supervised the project. All authors read and approved the final manuscript.
